# Step-Length Estimation in Asymmetric Gait Using a Single Lower-Back IMU Data and a Biomechanical Model Inspired by a Double Inverted Pendulum

**DOI:** 10.3390/bioengineering13010003

**Published:** 2025-12-20

**Authors:** Daniela Pinto, Paulina Ortega-Bastidas, Pablo Aqueveque

**Affiliations:** 1 Electrical Engineering Department, Faculty of Engineering, Universidad de Concepción, Concepción 4070386, Chile; danielapinto@udec.cl; 2 Kinesiology Department, Faculty of Medicine, Universidad de Concepción, Concepción 4070386, Chile; portegab@udec.cl

**Keywords:** human locomotion, gait analysis, wearable sensor, inverted double pendulum model, stride length

## Abstract

Step length is a fundamental parameter for gait assessment, reflecting complex neuromuscular and biomechanical behavior. Accurate step-length estimation is clinically relevant for monitoring populations with neurological or musculoskeletal conditions, as well as older adults. This study presents a novel biomechanical model, inspired by the inverted double pendulum, for step-length estimation under asymmetric gait conditions using a single inertial sensor on the lower back. Unlike models that assume symmetry, the proposed model explicitly incorporates pelvic rotation, enabling more accurate step length estimation, particularly in individuals with gait impairment. The model was validated against a gold standard OptiTrack^®^ (Corvallis, OR, USA) system with 33 adults: 21 participants without and 12 with gait impairment. Results show that the model achieved low Median Absolute Errors (MdAE), below 0.04 m in participants without gait impairment and remaining within 0.06 m in those with impairment. Statistical validation confirmed a strong correlation with the reference system (R = 0.96, R^2^ = 0.93) and a clinically trivial mean bias (0.64 cm) from Bland-Altman analysis. These results validate the model’s effectiveness under various gait conditions, suggesting its technical feasibility and strong potential for clinical and real-world applications, particularly for the longitudinal monitoring of patients with functional impairments.

## 1. Introduction

Step length is a fundamental spatial parameter for assessing motor function, as it allows evaluation of the interaction among the multiple systems involved in gait [1]. Its analysis is crucial for identifying impairments, guiding rehabilitation, and promoting a better quality of life in populations with neurological disorders or musculoskeletal conditions, or older adults [2]. In neurological conditions such as Parkinson’s disease, gait is often affected due to the progressive nature of the pathology, which leads to bradykinesia, tremors, rigidity, and reduced movement fluidity, all of which can impair step length and walking speed [3]. Conversely, in patients with stroke sequelae such as foot drop, gait is influenced by compensatory strategies. These include excessive hip and knee flexion or a circumduction pattern, in which the affected leg swings in a lateral arc to avoid dragging [4]. Such asymmetric patterns diminish gait efficiency, reduce walking speed [5], increase fall risk, and raise the energetic cost of locomotion [6,7].

Regarding older adults, gait plays a crucial role in health and functional independence. It is estimated that 13% of individuals between 65 and 69 years of age report balance problems, a figure that increases to 46% in those over 85 years old. Balance and gait impairments are key determinants of fall risk [8], stemming from the progressive decline in sensory function, central nervous system integration, and neuromuscular and skeletal capacity associated with aging. Moreover, the comorbidities prevalent in this population can exacerbate these impairments, and the medications prescribed for chronic conditions may further compromise stability and gait patterns [8]. Consequently, the interplay of these factors heightens both the risk of and fear of falling, leading to reduced physical activity and, in turn, decreased muscle strength, flexibility, and bone integrity [9]. This sequence undermines locomotor efficiency, slowing walking speed through shorter steps, thereby requiring more steps to traverse the same distance and increasing muscular demand. Additionally, fear of falling fosters compensatory gait adaptations, such as foot dragging, which limit transverse pelvic rotation and restrict pelvic mobility. The ensuing loss of postural control and sensorimotor deterioration can amplify gait asymmetry, further elevating fall risk and diminishing the capacity to recover from perturbations [8].

Thus, assessing step length is essential for detecting gait abnormalities and designing rehabilitation strategies. Various methods [2,10,11,12] have been developed, ranging from observational techniques to advanced technological systems. Among these, inertial sensors, or Inertial Measurement Units (IMUs), stand out for their ability to capture measurements without requiring controlled environments such as gait laboratories or optoelectronic systems. Their low cost, portability, and ease of use make them a viable alternative for gait analysis in diverse settings. Due to their high sensitivity, IMUs can precisely detect subtle changes in gait kinematics, enabling earlier identification of mobility impairments with greater efficiency than conventional methods. This makes them a practical and accessible tool for monitoring and analyzing gait in clinical, home, or community environments [13]. The versatility and accessibility of inertial sensors have driven the development of numerous models and algorithms for step length estimation, as proposed by various authors in the literature.

In 2015, the authors [14] recruited 80 adult volunteers and divided them into two groups of 40 participants: the first group consisted of healthy young adults aged 20 to 40 years, and the second comprised older adults aged 50 to 70 years. Each participant wore an inertial sensor sampling at 100 Hz, mounted at the level of the L5 vertebra on the lower back using a dual-strap belt. In addition, they walked over a GAITRite pressure platform sampling at 240 Hz while being recorded by a video camera at 25 fps. Participants were asked to walk at their self-selected speed for two minutes along a 25 m circuit. After data collection, the signals were processed in MATLAB 2012: gait segmentation was performed by applying a Wavelet Transform to the vertical acceleration to identify Heel Strike (Initial Contact, IC), and then the derivative of that signal was used to detect Toe Off (Final Contact, FC), corresponding to the minima and maxima of their respective acceleration profiles. Step length was computed using the simple inverted pendulum model described in [15], which relies on double integration of the vertical acceleration to estimate changes in the center-of-mass height combined with leg length. The results showed a mean step length of 78.63 ± 9.30 cm in young adults and 79.83 ± 9.80 cm in older adults. The authors concluded that, although the agreement between their calculated step lengths and those measured by the pressure platform and video system was good, concordance in variability, asymmetry, and left-versus-right step detection was poor—not because of sensor inaccuracy, but due to the formula’s inability to account for asymmetric gait patterns or center-of-mass tilts. This highlights the critical need for a model that, rather than assuming symmetry, can capture the individual dynamics of each limb.

Moe-Nilssen and colleagues [16], measured 23 subjects aged 20 to 49 years using an inertial sensor placed at the level of the L3 vertebra on the lower back, sampling at 128 Hz along a 6–9 m walk path, with length adjusted to the available space and individual. To estimate step length, they first preprocessed the acceleration data by correcting for the gravitational component to isolate the true dynamic acceleration. They posited that, during steady-speed walking, the net acceleration along each axis averages to zero, and that any nonzero mean acceleration arises from the average gravitational vector on that axis. Consequently, the raw measurements must be rotated into a horizontal–vertical coordinate frame via a trigonometric algorithm to recover true acceleration values. For step-length calculation, the authors invoke a useful corollary stating that the mean step length over a walking sequence can be determined from the sampling frequency and walking speed. Using this approach, they achieved a step-length resolution better than 0.008 m. However, they noted that their correlation-based system exhibits limited robustness with large data volumes, since postprocessing variance increases, and that a minimum number of steps is required for reliable estimation, constrained by the chosen sampling frequency.

Finally, the authors in [17] propose various methods for calculating step length and other spatiotemporal parameters using different configurations of inertial sensors, either a single sensor on the lower back or three sensors (one on the lower back and one on each calf). Focusing on the single-sensor approach, they report favorable outcomes across twelve methods grouped into four categories: biomechanics-based, double-integration-based, machine-learning-based, and hybrid combinations. However, only the integration-based methods represent novel contributions; the others consist of incremental modifications or were included solely for comparative purposes.

Regarding the integration-based methods, four new approaches are proposed: three rely on double integration of the acceleration signal with drift-error compensation inspired by the main ideas in [18] and the fourth takes the average of the other three. For the first step-length estimate (STPL5), the authors used the acceleration in the z-axis (anteroposterior), which they filtered with a second-order Butterworth filter having a 0.5 Hz cutoff frequency. They then performed double integration of the filtered signal between two successive initial contacts. Finally, to mitigate cumulative error, they calculated the mean acceleration over that same interval and incorporated it into the formula, as presented in Equation (1)

(1) $$STPL_{5}\left[m\right]=A\left(\underset{ic(m)\leq t\leq ic(m+1)}{\underset{︸}{\left|\max(p(t))-\min(p(t))\right|}}\times a_{\mathrm{apMean}}\left(m\right)\right)+B$$

where:

(2) $$a_{\mathrm{apMean}}\left(m\right)=\underset{ic(m)\leq t\leq ic(m+1)}{\mathrm{mean}}\left(a_{ap}\left(t\right)\right)$$

The second proposed method likewise employs double integration but incorporates Empirical Mode Decomposition (EMD) to remove drift. First, the authors subtract the mean value of the z-axis acceleration to prevent any initial bias from contaminating the integration process. They then perform a first integration to derive velocity and apply EMD, decomposing the velocity signal into its intrinsic mode functions (IMFs). By reconstructing the signal using only the first four IMFs, they effectively reduce noise and minimize drift introduced during integration. Next, they integrate the denoised velocity signal a second time to obtain position, apply EMD again, and reconstruct the signal from the first three IMFs. Finally, step length is computed using the same drift-compensated formula presented in Equation (1).

In the third method, the authors correct drift by initially applying the Zero Velocity Update (ZUPT) technique, which assumes zero velocity at each initial contact. However, this assumption does not hold when the IMU is mounted on the lower back, since the trunk can continue moving even while the foot is planted. To overcome this, they integrate the anteroposterior acceleration under the ZUPT assumption to obtain a linear velocity profile. In parallel, they compute the mean velocity for each gait cycle using Equation (3) and use this value to correct the linear velocity: for each step, they replace the velocity’s mean with the calculated mean, yielding a drift-corrected velocity time series. Finally, this corrected velocity is integrated to derive position, from which step length is calculated via Equation (8). It should be noted that, across all methods, the variables A and B serve as correction coefficients optimized during model training.

(3) $$V_{\mathrm{mean}}\left[m\right]=\left(\frac{STPL_{1}\left(m\right)+STPL_{2}\left(m\right)}{2}\right)\times CAD\left(m\right)$$

(4) $$STPL_{1}\left[m\right]=A\left(2\sqrt{2ld_{\mathrm{step}}\left[m\right]-d_{\mathrm{step}}{\left[m\right]}^{2}}\right)+B$$

(5) $$d_{step}\left[m\right]=\underset{ic(m)\leq t\leq ic(m+1)}{\underset{︸}{\left|\max\left(d_{v}\left(t\right)\right)-\min\left(d_{v}\left(t\right)\right)\right|}}$$

(6) $$STPL_{2}\left[m\right]=A\left(\sqrt[{4}]{a_{\mathrm{maxmin}}\left(m\right)}+B\right)$$

(7) $$a_{\mathrm{maxmin}}\left(m\right)=\underset{ic(m)\leq t\leq ic(m+1)}{\underset{︸}{\left|\max\left(a_{v}\left(t\right)\right)-\min\left(a_{v}\left(t\right)\right)\right|}}$$

where $a_{v}$ is the vertical acceleration of the center of mass, CAD: cadence

(8) $$STPL_{7}\left[m\right]=A\left(\max_{ic(m)\leq t\leq ic(m+1)}\left(p\left(t\right)\right)\right)+B$$

The authors evaluated 40 adult participants, both with and without gait pathologies, with a mean age of 62 ± 8 years. Data collection involved an inertial sensor mounted on the lower back sampling at 128 Hz, alongside a pressure platform also at 128 Hz used as the reference standard. The acquisition protocol required participants to walk 12 m at their self-selected speed for one minute; they were then instructed to increase or decrease their walking speed, and assistive devices were permitted if needed. From their results, the authors concluded that combining multiple estimation methods yielded greater precision in step-length measurement than using any single method alone. Specifically, biomechanical models outperformed other approaches during slow walking, achieving an RMSE of 0.04 m, owing to their reliance on principles such as the inverted-pendulum model rather than solely on acceleration signal patterns as with integration-based methods (which exhibited an RMSE of 0.07 m). Under normal walking conditions, integration-based algorithms attained the best performance, with an RMSE of approximately 0.08 m, while during fast walking no significant differences emerged among the evaluated approaches. Furthermore, in participants using assistive devices, both machine-learning and biomechanical models demonstrated greater robustness due to the increased variability in their movements. Finally, the authors emphasized that accurate detection of initial contact events significantly influences the overall performance of step-length estimation algorithms.

Finally, the authors emphasized that the precise detection of initial contact events significantly influences the overall performance of the algorithms. This dependency, coupled with the fact that existing models lose robustness under challenging conditions, highlights the need for an approach that is robust by design. However, accuracy, the reliance on symmetry and the simplification of dynamics in current models is not a minor technical detail; it is a barrier with direct clinical consequences. In a patient with asymmetrical gait, such as in cases of hemiparesis, the body uses pelvic rotation as a fundamental compensatory mechanism to advance the affected limb. Therefore, models that ignore this rotation can erroneously interpret this compensatory strategy as functional step length, leading to a critical underestimation of the actual asymmetry.

Translating this to a clinical setting, a therapist might mistakenly conclude that a patient’s gait is improving when, in reality, a compensatory pattern is becoming entrenched. This can lead to incorrect therapeutic decisions or an imprecise assessment of rehabilitation progress, hindering the objective monitoring of recovery. It is precisely this need for a biomechanically faithful measurement that motivates our work.

To overcome this barrier, this work presents a biomechanical model based on a inverted double-pendulum. The contribution of our approach, therefore, is not an abstract improvement but a direct response to these clinical shortcomings. It offers superior biomechanical fidelity by modeling each limb independently and explicitly incorporating pelvic rotation, thus avoiding clinical misinterpretations. Furthermore, it provides improved robustness, demonstrating consistent performance under varied conditions and overcoming the limitations of methods that report significant errors. Finally, its clinical viability is achieved by using a single inertial sensor, resulting in an accessible tool for the longitudinal monitoring of patients in their own environment.

Thus, our model is not merely an incremental improvement, but an advance toward a more precise, robust, and clinically applicable gait assessment.

## 2. Materials and Methods

**Figure 1:**
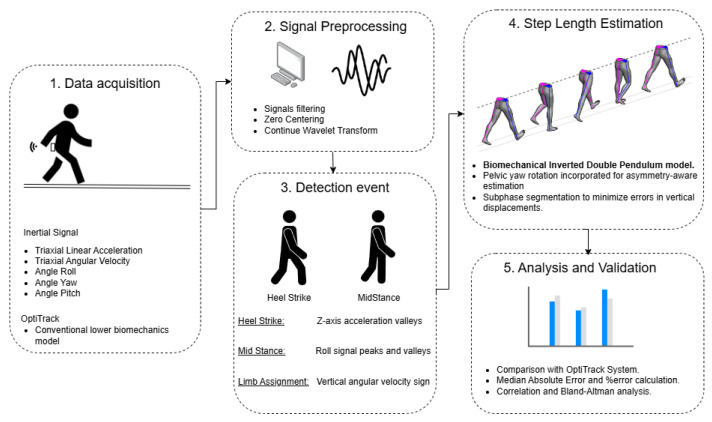
Methodology used in this study divided into 5 stages: Data acquisition, Signal Preprocessing, Detection event, Step Length Estimation and Analysis and Validation.

This section presents the methodology used in this study, as illustrated in Figure 1. The process begins with data acquisition, followed by signal preprocessing, gait event detection, step length estimation, and finally, analysis and validation of the results using the OptiTrack^®^ system as the gold standard.

### 2.1. Participants

Thirty-three adults voluntarily participated in the study and were classified into two main groups: with and without gait impairment. Participants with impairment were assigned to subgroups DA, DB and DC, while those without impairment were classified into subgroups A, B, and C. Subgroup assignment was based on age range, as detailed in Table 1. All participants provided informed consent approved by the Committee on Ethics, Bioethics, and Biosafety of the Vice-Rectory for Research and Development at the Universidad de Concepción (approval code CEBB 1757-2024).

All participants underwent an initial clinical evaluation conducted by a licensed physiotherapist before data collection. This assessment included measurement of blood pressure, oxygen saturation, and anthropometric parameters, as well as administration of the Berg Balance Scale and documentation of fall history within the previous year. Cognitive function was also evaluated in all participants using the Mini-Mental State Examination (MMSE). Participants were included if they were over 18 years of age and capable of walking with or without assistive devices. Exclusion criteria comprised inability to follow simple commands, intolerance to physical effort, decompensated medical conditions, history of lower-limb or trunk surgery within the last two years, or musculoskeletal pain in the lower limbs greater than 5 on the Numerical Analog Scale (NAS).

Additionally, and exclusively for algorithmic validation purposes, recordings were excluded when gait events such as initial contact or mid-stance were clinically absent, as confirmed by the physiotherapist through direct observation on OptiTrack signals. These cases corresponded to participants who did not exhibit a distinguishable stance-to-swing transition, making step segmentation unfeasible in the current implementation. This condition does not represent a clinical exclusion criterion but rather a technical limitation of the algorithm, which currently requires identifiable gait events for step segmentation.

Participants with gait impairment presented verified neurological or musculoskeletal conditions that affect gait symmetry or pelvic control. The specific diagnoses were:

- Vertigo disorder.
- Parkinson’s disease
- Osteoarthritis.
- Traumatic brain injury (TBI).
- Stroke.

In addition to the general evaluation, participants with gait impairment underwent a specific neuromusculoskeletal assessment performed by the physiotherapist, which included examination of joint range of motion, muscle tone, sensitivity, proprioception, and muscle strength.

### 2.2. Instrumentalization and Experimental Protocol

An optoelectronic motion capture system (OptiTrack^®^, 12 cameras), considered the gold standard for gait analysis, and a single inertial measurement unit (IMU) mounted on the lower back were used concurrently to collect kinematic data.

**Figure 2:**
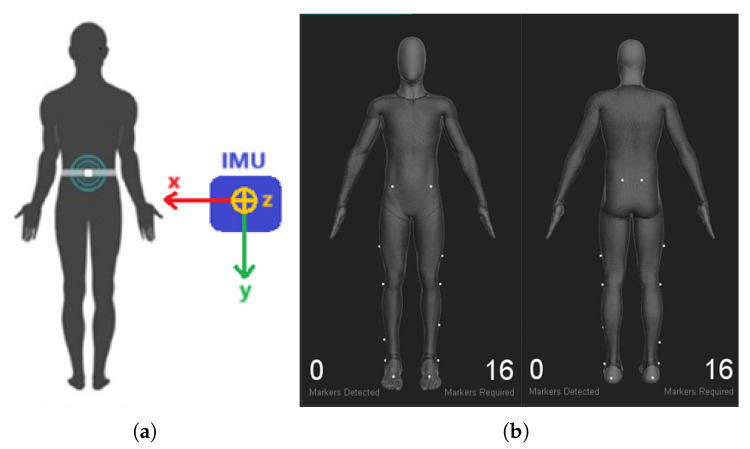
Participant Instrumentation. (**a**) Inertial sensor placement. (**b**) Marker placement according to the “Conventional Lower” biomechanical model of OptiTrack^®^.

The lower-back placement has been shown to be optimal for detecting gait events due to its proximity to the body’s center of mass [19,20]. The IMU was secured with an elastic belt, adjusted for each participant’s comfort to minimize movement artifacts and maintain consistent alignment throughout the trial (Figure 2a).

Sixteen passive markers were placed on the lower limbs according to the Conven-tional Lower Biomechanical Model (Figure 2b) to enable precise tracking of joint motion and segment positions, consistent with standard clinical gait protocols [21].

Participants performed the 10-Meter Walk Test (T10M) three times, as recommended in gait assessment studies, to account for intra-subject variability and obtain representative measurements of typical gait patterns [22]. All trials followed standardized instructions, with participants walking at a self-selected comfortable pace, allowing reliable comparison between IMU-based estimations and the optoelectronic reference.

A homemade inertial sensor developed for this study incorporates a three-axis accelerometer, a three-axis gyroscope, and a three-axis magnetometer (Table 2). It features an onboard processor that fuses magnetic and inertial data via an extended Kalman filter to output orientation in quaternion form, thereby avoiding the singularities inherent to Euler angles and navigation angles. Orientation data are provided in angular representation with an accuracy of ±1°. Sensor readings are sampled at 100 Hz and transmitted via Bluetooth 5.0 [23]. The robustness evaluation of this fusion demonstrated a low Dynamic Yaw Error (0.25° ± 0.36°) in stable environments [24].

### 2.3. Identification of Gait Events with OptiTrack System

After post-processing the signals acquired with the OptiTrack^®^ system, the data were analyzed using Motion Kinematics and Kinetic Analyzer (Mokka^®^), version 0.6.2.0, 2013. A physiotherapist identified the initial contact, mid-stance, and toe-off events for both lower limbs using pelvis, knee, and heel markers to ensure precise and consistent identification. The IMU events were detected separately through the proposed method.

To synchronize both data streams, the first heel contact of each trial—independently identified in both systems—was used as a temporal reference. From this common reference point, step duration and step length were computed in parallel to evaluate the performance of the IMU-based method relative to the OptiTrack^®^ reference.

## 3. Model Proposed: Step Length Estimation

**Figure 3:**
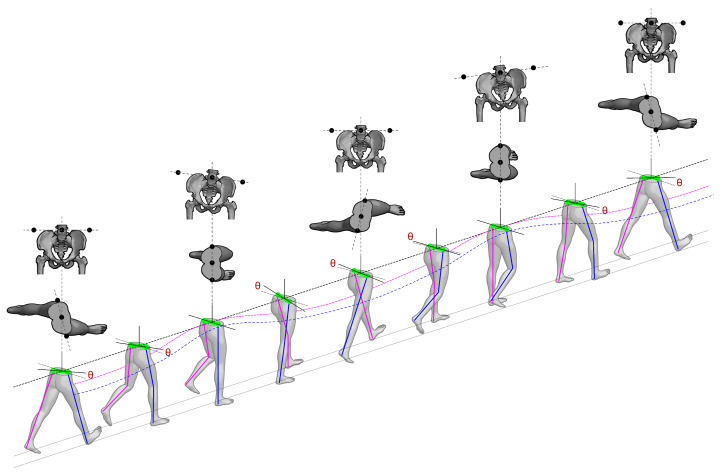
Biomechanical schematic inspired by the double inverted-pendulum, where both legs act as independent inverted pendulums connected through the pelvis. The pelvis functions as the common coupling body, transmitting motion and energy between limbs and contributing to overall step generation. The pelvis (green), right leg (blue), and left leg (pink) are shown; pelvic rotation relative to the horizontal plane is quantified by the angle θ (red) and dotted lines indicate the vertical displacement for each inverted pendulum (blue and pink).

Given that gait biomechanics is largely determined by body structure [25], we propose an evolution of classical models: the biomechanical inverted double pendulum. In this model, each limb is treated as an independent pendulum connected to the pelvis, which acts both as a linking body and as the center of mass, as shown in Figure 3. This configuration allows the model to capture human gait dynamics more realistically, without assuming symmetry between the body’s sides. The model is capable of representing the nonlinear nature of locomotion and simulating leg dynamics [26], making it particularly relevant for individuals with gait impairments, where the behavior of the center of mass (CoM) may become irregular or exhibit compensatory displacements, ultimately affecting locomotor efficiency [27].

Furthermore, the pelvis, serving as the link between the two pendulums, transmits forces that maintain balance and stability during gait and functions as the support base for the legs. Its position and orientation vary to optimize displacement efficiency and determine step length. Therefore, movements must be considered not only in the sagittal plane but also in the frontal and transverse planes. Specifically, pelvic tilt toward the stance leg in the frontal plane and counter-rotation of the swinging leg in the transverse plane are key to understanding overall displacement and ensuring smooth transitions between steps [25]. During a gait cycle, transverse-plane pelvic rotation generates longitudinal displacement when the pelvis alternately rotates opposite to the swinging leg (Figure 3). Insufficient rotation reduces step length and walking efficiency, whereas excessive rotation compromises stability and increases the risk of falls.

Therefore, based on this biomechanical rationale, our model decomposes step length ($L_{dP}$) into two main components that reflect these dynamics. This approach allows us to present the model’s structure conceptually before detailing its full mathematical derivation in Equation (9)

(9) $$L_{dP}=L_{pendular}+L_{pelvic}$$

where: $L_{pendular}$ represents the pendular component of the step. This is the baseline distance generated by the motion of the leg acting as an inverted pendulum, and it is the component that simpler models attempt to capture. $L_{pelvic}$: Represents the rotational pelvic component. This term is our key contribution to capturing asymmetry and represents the additional (or subtracted) length generated directly by the rotation of the pelvis in the transverse plane—a factor that, as we have discussed, is crucial for accurate estimation.

Taken together, these two terms reflect not only the displacement of the leg but also the coupling between both limbs through the pelvis. From a biomechanical standpoint, the proposed model is therefore based on a parallel coupled inverted double-pendulum configuration, in which each leg behaves as an independent inverted pendulum during its stance phase. Both pendulums share a common body—the pelvis—that serves as a dynamic coupling element, transmitting motion and energy between limbs. The pelvic rotation in the transverse plane produces the additional displacement modeled by the $L_{pelvic}$ term, thus representing the interaction between both pendulums and their shared body. This formulation provides a faithful representation of human gait dynamics, where each step emerges from the coordinated coupling between pelvic oscillations and alternating stance phases of the lower limbs.

This equation represents the complete formulation of our biomechanical model, where the total step length ($L_{dP}$) is determined by the synergy of its two primary constituents:

### 3.1. Pendular Component

**Figure 4:**
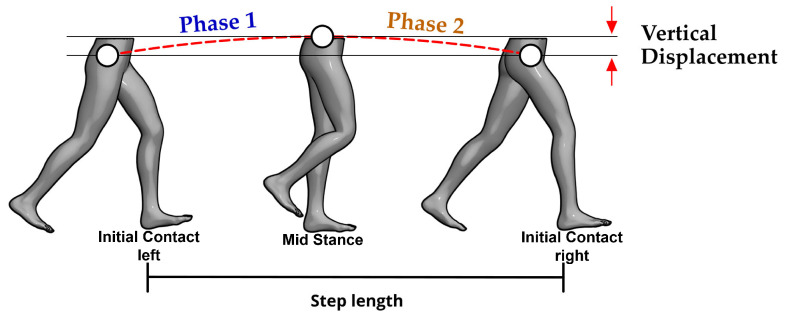
Selection of stages for event detect.

To reduce measurement errors, it must be noted that a human step is not a monolithic motion. From the perspective of an inverted pendulum (Figure 3), the dynamics change crucially depending on whether the body’s center of mass (located in the pelvis) is behind or in front of the supporting foot. For this reason, our model divides each stance phase into two sub-phases (Figure 4):

- Phase 1 (*E*1—Acceleration/Deceleration): Extends from the initial contact until the pelvis is di-rectly over the ankle. In this phase, the body is braking its forward fall.
- Phase 2 (*E*2—Propulsion): Spans from the moment the pelvis passes the ankle until the next initial contact. Here, the body is propelling itself forward.

This division is key to capturing asymmetry, as it allows the duration and dynamics of each phase to differ for the left and right legs, thus reflecting the compensations that occur in pathological gait.

To mathematically describe the dynamics of these two phases, it is necessary to characterize the displacement of the center of mass. A fundamental principle of inverted pendulum dynamics is that the forward velocity is intrinsically linked to the individual’s cadence and leg length. This fundamental relationship allows us to establish an initial description of the motion, as shown in the Equation (10)

(10) $$V=C\cdot L_{pendular}$$

where *V* is velocity, *C* is cadence and $L_{pendular}$ is step length.

The formulation of our step length model is inspired by the Inverted Pendulum (IP) principle, which assumes two key physical conditions: (1) the supporting leg acts as a rigid bar pivoting on the foot’s contact point, and (2) the Center of Mass (CM) describes a circular arc under the approximate conservation of energy during single support. However, for practical implementation and clinical robustness, we chose a kinematic and geometric approximation (Equations (10)–(13)) instead of the second-order differential equations that define the complete double pendulum dynamics. This choice was a strategic design decision. The kinematic formulation allows us to achieve a computationally efficient solution and, more importantly, one that is stable against acceleration integration error.

**Figure 5:**
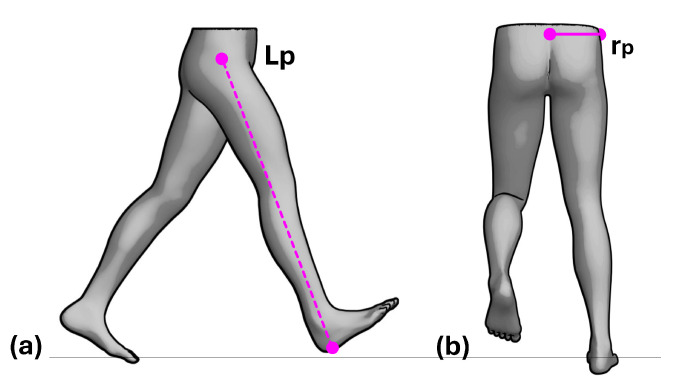
(**a**) $L_{p}$ defined as the distance from greater trochanter to the sole of the foot. (**b**) $r_{p}$ defined as the distance from the midpoint of the sacrum to the iliac crest.

Then, for this relationship to be robustly applicable across a diverse population, the model must be independent of the individual’s scale (i.e., their height and specific walking speed). For this purpose, we introduce the Froude number (*Fr*), a dimensionless parameter well-established in gait analysis that normalizes walking dynamics [28]. The Froude number provides a more universal description of gait by relating the inertial forces (associated with movement) to the gravitational forces (associated with the individual’s height and leg length, as shown in Figure 5a) as shown in Equation (11). This allows us to analyze the dynamics in a scalable and comparable manner between subjects.

(11) $$Fr=\frac{V^{2}}{g\cdot L_{p}}$$

where *Fr* is Froude number, *V* is velocity, g gravity and $L_{p}$ is leg length.

Therefore, by substituting (10) into (11), we obtain (12)

(12) $$Fr=\frac{{\left(C\cdot L_{\mathrm{pendular}}\right)}^{2}}{g\cdot L_{p}}$$

And if we solve for $L_{pendular}$ we get (13):

(13) $$L_{\mathrm{pendular}}=\frac{\sqrt{F_{r}\cdot g\cdot L_{p}}}{C}$$

We acknowledge that the Froude Number (Fr) is traditionally used for constant velocities over the full stride. However, our use of phase-specific Froude numbers ($Fr_{E1}$ and $Fr_{E2}$) is a direct consequence of the need to decompose the Center of Mass (CM) dynamics in pathological gait. In these subjects, the Braking phase (E1) and the Propulsion phase (E2) are not symmetric in either time or velocity. Using a single Fr would force a constant average velocity that would obscure the kinematic differences between how the subject initiates the step and how it ends. Therefore, the Fr in each subphase is computed from the integrated average velocity (*V*) of that segment and serves as a dimensionless scaling parameter that allows us to represent the true dynamical differences in time and space between the two subphases. In summary, phase-specific Fr enables us to transform the two distinct segmented kinematic velocities ($V_{E1}$, $V_{E2}$) into a comparable and robust factor (Equation (13)), which is essential for the subsequent analysis of asymmetry.

### 3.2. Pelvic Component

This term represents our direct solution to the main limitation of traditional pendulum models: their inability to account for pelvic rotation. Our model, in contrast, explicitly quantifies how pelvic rotation in the transverse plane generates an additional displacement component—a factor that is crucial for correctly estimating step length under real-world and asymmetrical gait conditions.

(14) $$L_{\mathrm{Pelvic}}=r_{p}\cdot \tan{\left(\mathrm{yaw}(t)\right)}_{E1,E2}$$

where $r_{p}$ is the pelvic radius, and yaw(*t*) is the pelvic rotation over the time interval during which step length is measured (Figure 5b). *E*1 denotes Phase 1, from the initial contact of one foot to mid-stance, and *E*2 denotes Phase 2, from mid-stance to the initial contact of the contralateral foot (Figure 4).

It is important to note that the choice of the function tan(yaw(t)) in Equation (14) is grounded in the geometric projection on the transverse plane, assuming pelvic motion as a rotation around a vertical axis. When modeling the pelvis from a top view, the effective pelvic radius ($r_{p}$), is represented as the adjacent side of the projection triangle. The longitudinal displacement of the pivot point in the forward direction ($L_{\mathrm{pelvic}}$) is modeled as the opposite side resulting from the rotation yaw(t).

While the small-angle approximation (θ o sin(θ)) is a common convention in non-pathological gait, the full tan(θ) formulation is geometrically more accurate and was chosen to preserve the validity of the projection across the larger compensatory angles observed in impaired gait. Furthermore, we confirm the numerical stability of this component: the maximum range of pelvic rotation (Yaw) observed across our entire cohort, including pathological gait, was contained within $\pm 20^{\circ }$, which is safely remote from the singular points ($\pm 90^{\circ }$) of the tangent function.

$L_{pelvic}$ term has a direct geometric interpretation: it represents the lateral contribution to step length generated by the rotation of the pelvis in the horizontal plane. It is, in essence, the component of the step that originates not from the pendular motion of the leg, but from the rotation of the pelvis, making it fundamental for correcting estimations in asymmetrical gait.

Finally, by integrating the components we have developed, we arrive at the complete formulation of our model. Step length ($L_{dP}$) is defined as the sum of the pendular displacement (calculated through its braking and propulsion phases) and the rotational contribution of the pelvis. This unification results in the model’s final Equation (15)

(15) $$L_{dP}=\left(\frac{\sqrt{F_{rE1}\cdot g\cdot L_{p}}}{C_{E1}}-r_{p}\cdot \tan{\left(\mathrm{yaw}(t)\right)}_{E1}\right)+\left(\frac{\sqrt{F_{rE2}\cdot g\cdot L_{p}}}{C_{E2}}-r_{p}\cdot \tan{\left(\mathrm{yaw}(t)\right)}_{E2}\right)$$

$L_{dP}$ represents more than a simple formula, it is a biomechanically complete model. By capturing the individual dynamics of each leg and the direct influence of the pelvis, it stands as a functional representation of human gait. It is precisely this capacity to incorporate the mechanisms of asymmetry that establishes it as a robust tool for precise quantification in clinical analysis.

However, the practical application of this model is contingent upon a rigorous signal processing and analysis framework. The following section (Section 4) is dedicated to detailing this crucial step. It outlines the methodology used to translate the raw sensor outputs into the specific biomechanical variables required by our model, and subsequently, the protocol used to validate their accuracy. Therefore, this next section provides the essential link between theoretical formulation and its practical, validated application.

## 4. Signal Processing and Data Analysis

All signal processing was conducted in MATLAB^®^ R2024a. Our methodology is built upon two key components: the detection of discrete gait events and the continuous tracking of pelvic orientation. As these components have distinct requirements, they are processed using different data streams and reference frames from the inertial sensor.

The detection of gait events, such as initial contacts and the differentiation between limbs, relies on the sharp, transient patterns found in the raw accelerometer and gyroscope signals. These are most effectively analyzed in the sensor’s local (body-fixed) reference frame and are therefore used without transformation. In contrast, tracking pelvic rotation requires stable spatial orientation, which is derived from the sensor’s fused data in a global reference frame.

The following subsections detail the specific procedure used for each component, beginning with the identification of gait events.

### 4.1. Identification of Gait Events

To detect initial contact, we selected the anteroposterior acceleration. At the moment the foot strikes the ground, the pelvis undergoes a rapid deceleration, which appears as a characteristic valley in this same anteroposterior acceleration signal; this event repeats each gait cycle, allowing consistent identification [29].

To detect this valley, we first applied a spectral analysis to select the frequencies to filter basing in the Continuous Wavelet Transform (CWT) to the anteroposterior acceleration signal in order to obtain a time-frequency representation and identify the dominant frequency band associated with gait. In performing this analysis, we prioritized frequency resolution to accurately identify the signal’s power band, accepting the lower temporal resolution inherent in the analysis of low frequencies.

This analysis confirmed that the fundamental frequency of locomotion was consistently found in the 1.5 to 1.8 Hz range. Based on this finding, the following signal processing pipeline was defined and applied to the acceleration signal:

First, a 4°-order Butterworth high-pass filter with a cutoff frequency of 0.5 Hz was applied. The purpose of this filter was to remove the signal offset.

Second, a 4°-order Butterworth low-pass filter with a cutoff frequency of 1.8 Hz was applied. The goal of this filter was to isolate the fundamental acceleration/deceleration wave. In this way, steps could be identified, which would lead to initial contact detection.

Next, we applied the detection algorithm. Since the deceleration valley (IC) is reliably preceded by the peak acceleration of the step, our method first locates this peak. We found these peaks by computing the signal’s time derivative and identifying its zero-crossings, applying an adaptive threshold to distinguish peaks. After identifying a peak, we defined a search window of four samples before and after to locate the local minimum (the valley). This minimum was registered as the initial contact. We chose a ±4-sample window (at 100 Hz) as it offered the best balance of stable detection and temporal accuracy after testing multiple window sizes. This fixed-size window was selected for its contribution to algorithmic stability and for meeting the requirements of low computational complexity and low latency. It demonstrated the highest stability in identifying the acceleration valley across the wide spectrum of gait patterns evaluated.

For mid-stance detection, we turned to the frontal-plane rotation (roll) signal, which reflects pelvic tilt as load shifts between limbs. Depending on whether the stance leg is on the left or right, this rotation produces either a maximum or a minimum in the roll trace, providing a clear marker. We first high-pass filtered the roll signal to center it around zero, then took its derivative and used zero-crossing detection to locate the relevant peaks and valleys.

Finally, to determine which limb is in stance at each initial contact, we examined the vertical-axis angular velocity. Immediately following an initial contact event, a positive angular velocity indicates that the center of mass is moving to the right implying the left leg is in support whereas a negative value indicates movement to the left and right-leg support.

### 4.2. Pelvic Rotation

For pelvic rotation (yaw(*t*)), which requires a stable spatial orientation, we used the stream of quaternions provided by the sensor. These quaternions represent the sensor’s orientation in a global reference frame, with its vertical axis aligned with gravity. In post-processing, they were converted to Euler angles. The resulting yaw angle, which represents the rotation of the pelvis in the horizontal plane, was used as the primary input for our model’s pelvic component.

To ensure the stability of this signal and remove its offset, a custom high-pass filter was applied directly in the frequency domain. Therefore, to eliminate the offset (0 Hz component) and any low-frequency oscillations without affecting the gait signal, we conservatively chose to set the first eight frequency components to zero. This procedure effectively creates a high-pass filter with a cutoff frequency of approximately 0.25 Hz. The signal was then reconstructed using the inverse FFT (IFFT). Finally, a cross-correlation and circular shift were applied to the resulting signal to correct for any phase shift introduced by the filtering process.

This filtered yaw angle, combined with the pelvic radius ($r_{p}$), allows for a geometric estimation of the forward displacement contributed by the pelvis through the tangent function, as detailed in our model’s final Equation (15).

### 4.3. Integration of the Acceleration

Because linear velocity along the anteroposterior axis is required, the acceleration signal in that direction must be integrated. We therefore employ the OFDRI method (“Optimally Filtered Direct and Reverse Integration”) [20], which enhances integration accuracy by combining forward and backward integration with optimal filtering. This method was selected because it minimizes drift accumulation inherent to double integration while preserving the main kinematic components of gait. Compared with conventional numerical integration, OFDRI provides smoother and more stable velocity profiles over short walking trials, as previously demonstrated by [17].

First, the anteroposterior acceleration is low-pass filtered using a second-order Butterworth filter with a 3.5 Hz cutoff. This cutoff was chosen because biomechanical studies indicate that the center of mass exhibits significant components between 0.5 and 5 Hz, while muscle activity and high-frequency disturbances lie above 10 Hz [18]. The specific value of 3.5 Hz was determined empirically as the frequency providing the best balance between signal preservation and drift reduction. Lower cutoffs (<3 Hz) tended to attenuate peak accelerations, whereas higher ones (>4 Hz) introduced residual noise and instability during integration. The 3.5 Hz cutoff therefore yielded the most stable velocity reconstruction, aligning with the spectral bandwidth reported for pelvic and trunk motion during normal and pathological gait (typically below 4 Hz [30]).

First, the initial contact instants for the left and right limbs were identified, and the phases were segmented to begin forward integration. The trapezoidal rule was used for integration, with the initial velocity for Phase 1 (*E*1) set to zero corresponding to the instant when the foot lands. For Phase 2 (*E*2), the initial velocity was taken as the final velocity recorded at the end of *E*1, which occurs when the foot is vertical on the ground. For subsequent strides, each *E*1 phase’s initial velocity was set to the final velocity of the preceding stride. For reverse integration, the same phase segmentation was applied: the final velocity for each *E*1 and *E*2 was taken as the last velocity obtained from the forward integration of that phase. Finally, the combined velocity at each time point was computed by blending forward and reverse integrations according to equation

(16) $$V_{\mathrm{combined}}\left(t\right)=\alpha \cdot v_{\mathrm{direct}}\left(t\right)+\left(1-\alpha \right)\cdot v_{\mathrm{inverse}}\left(t\right)$$

where $V_{\mathrm{combined}}$ is the final velocity and α is the weighting factor.

Considering the previous equation, a weighting factor α of 0.5 was chosen so that both segments are given equal importance when fusing the forward and reverse velocities. To counteract the time-accumulated integration error, we apply a boundary-condition correction to ensure a stable signal consistent with gait patterns. Specifically, we perform a linear adjustment to distribute the velocity errors evenly over the entire trajectory. To achieve this, we use the following Equation (17)

(17) $$V_{\mathrm{corrected}}\left(t\right)=V_{\mathrm{combined}}\left(t\right)-\frac{t-t_{0}}{t_{f}-t_{0}}\cdot E_{v}$$

where $E_{v}$ is the velocity error.

To calculate $E_{v}$ a boundary value must be known to determine the target velocity; however, one cannot assume zero, since that assumption only holds when the sensor is placed on the foot where zero velocity at ground contact is valid. With the sensor on the lower back, the trunk may still be in motion despite foot contact, so the end-point velocity is not necessarily zero. Therefore, $E_{v}$ must be estimated or its end-point error minimized. In this case, because the true final velocity is unknown, it was approximated using the mean velocity calculated from Equation (16). Once the final velocity estimate is obtained, the Froude number for each phase is calculated by summing the velocities over that phase and using each participant’s leg length to compute step length via Equation (15).

### 4.4. Data Analysis

To rigorously evaluate the performance of our IMU-based model, its step length estimates were compared against those obtained from the OptiTrack^®^ motion capture system, which served as the gold standard due to its high precision in a controlled laboratory environment.

Our validation quantified the estimation error on a step-by-step basis. First, for each individual step, the Absolute Error (AE) was defined as the difference between the model’s estimate and the corresponding gold standard measurement, following (18).

(18) $$\mathrm{AE}=\left|{\mathrm{value}}_{\mathrm{calculated}}-{\mathrm{value}}_{\mathrm{estimated}}\right|$$

The relative error (% error) was also calculated to normalize the error against the true step length, following (19):

(19) $$\%\;\mathrm{error}=\frac{{\mathrm{value}}_{\mathrm{estimated}}-{\mathrm{value}}_{\mathrm{calculated}}}{{\mathrm{value}}_{\mathrm{calculated}}}\times 100$$

## 5. Results

**Figure 6:**
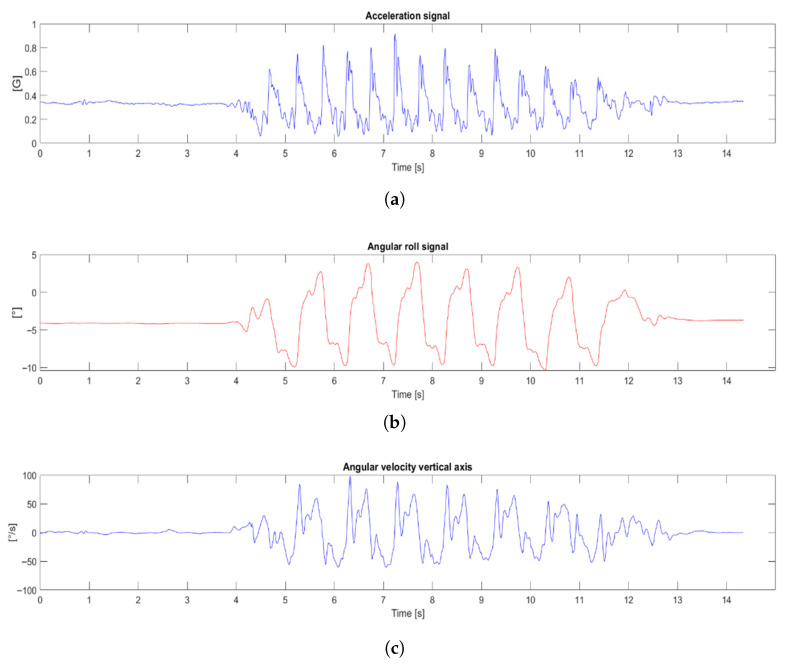
Signals used for gait event identification using an inertial sensor on the lower back. (**a**) Z-axis acceleration signal. (**b**) Roll angle signal. (**c**) Vertical angular velocity signal.

To evaluate the performance of the proposed inspired by the inverted double-pendulum model for step length estimation, the signals recorded from the IMU were analyzed to identify key gait events. Based on these detected events, subsequent analyses were performed to estimate step duration and length in both participants with and without gait impairment. The estimated parameters were compared with the OptiTrack^®^ system, the reference standard, and the resulting errors, variability, and agreement are presented in the following subsections. The z-axis acceleration signal (Figure 6a) is analyzed for initial contact detection the roll signal (Figure 6b) for mid-stance evaluation, the yaw signal for longitudinal displacement, and the vertical-axis angular-velocity signal (Figure 6c) to determine whether each contact corresponds to the right or left limb, as shown in Figure 6. In the acceleration signal, one can observe the number of steps taken by the participant and, as the pelvis undergoes a rapid deceleration, each of these instances can be identified as an initial contact an event that recurs every gait cycle, allowing for consistent detection [29]. The roll signal, by contrast, makes it possible to identify mid-stance events: as the pelvis tilts toward the stance leg, it generates an oscillatory waveform whose frequency matches the step count. Finally, the vertical-axis angular velocity distinguishes between limbs, since the pelvis oscillates side-to-side; by examining whether the value at initial contact is positive or negative, we can determine if it corresponds to the right or left foot.

**Figure 7:**
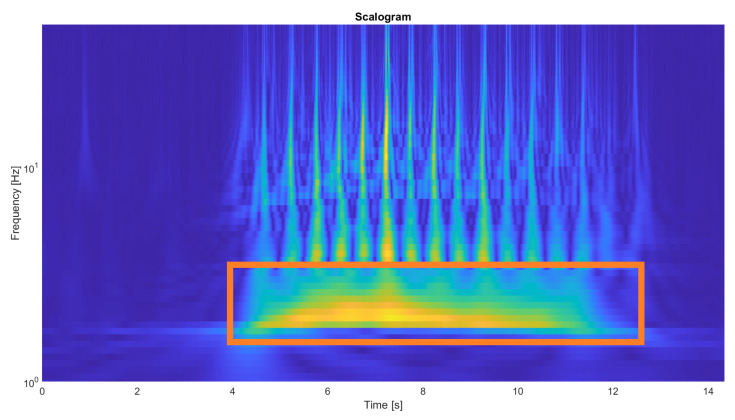
Spectrogram of the z-axis acceleration signal for step detection: the orange box represents the fre-quencies of interest.

**Figure 8:**
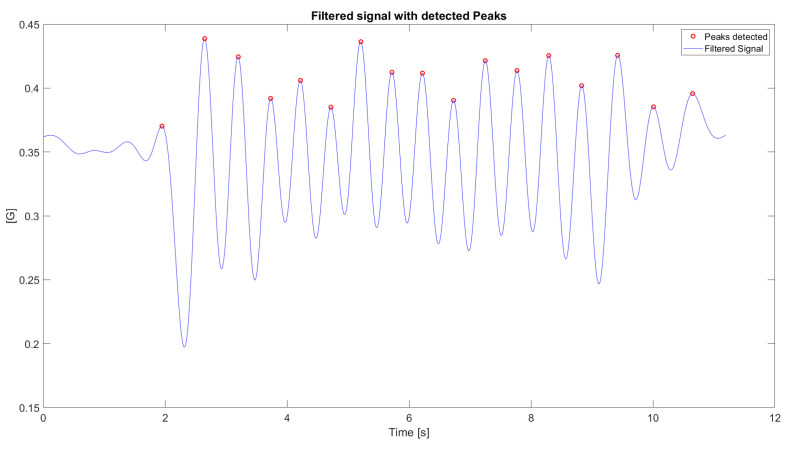
Step detection from the filtered z-axis acceleration signal where each detected peak corresponds to an initial contact event (step).

Regarding event detection, both initial contacts and mid-stance points were successfully identified for all subjects. In Figure 7, the spectrogram of a randomly selected participant is shown, with a box highlighting the frequency band chosen for filtering. Figure 8 shows the detected steps marked in red in the filtered signal, which will be used to detect initial contacts.

**Figure 9:**
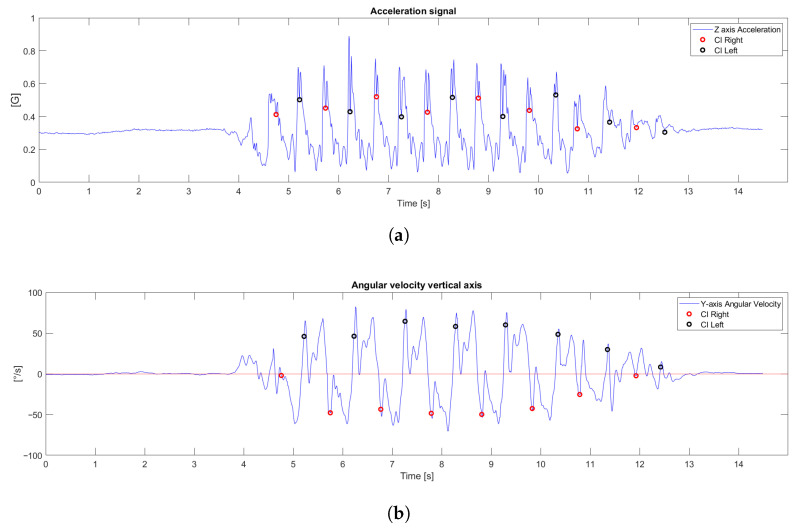
Gait event detection: (**a**) Initial contact detection using the z-axis acceleration signal. (**b**) Limb differentiation using the vertical-axis angular velocity.

Figure 9a illustrates the outcome of our event detection procedure for a representative subject. It displays the initial contacts, detected on the z-axis acceleration signal using the ten nearest-neighbor technique. Furthermore, the Figure 9b demonstrates the differentiation between the right and left limbs, which is achieved by analyzing the vertical angular velocity at the instance of each detected initial contact.

### 5.1. Initial Contact Detection

All initial contacts were successfully detected in all subjects, and it was successfully differentiated whether the initial contact was from the right or left limb.

### 5.2. Step Duration

To analyze the step durations and errors in step length estimation, the first and last two steps were excluded, as they represent the acceleration and deceleration phases of gait. Table 3, Table 4 and Table 5 present the step duration of participants from Groups A, B, and C, respectively, compared to the values obtained using the OptiTrack^®^ system.

Table 6, Table 7 and Table 8 show the step duration of participants with gait im-pairment from Groups DA, DB and DC, also in comparison with the OptiTrack^®^ system.

Given that the error distributions were found to be non-normal (Shapiro-Wilk test, *p* < 0.001), results are reported using non-parametric statistics: Median Absolute Error (MdAE) and Interquartile Range (IQR). The proposed model demonstrates consistent performance with low Median Absolute Errors across most subjects.

The results indicate that the proposed procedure performs well in adults without gait impairment. In Group A (older adults, Table 3), the median step duration error was 0.015 s (IQR: [0.010–0.018]) for the right foot and 0.010 s (IQR: [0.010–0.024]) for the left foot. In Group B (middle-aged adults, Table 4), the median error was 0.012 s (IQR: [0.010–0.015]) for the right foot and 0.010 s (IQR: [0.010–0.015]) for the left foot. In Group C (young adults, Table 5), the median error was 0.015 s for the right foot (IQR: [0.010–0.050]) and 0.015 s for the left foot (IQR: [0.010–0.025]). Overall, the median errors remained low and stable, though a notably higher gait variability was observed in the right foot of Group C.

In groups with gait impairment (DA, DB, DC), the results show that the procedure remains effective, although with greater error dispersion.

In Group DA (older adults with impairment, Table 6), the Grand Median error was 0.020 s (IQR: [0.010–0.030]) for the right foot and 0.020 s (IQR: [0.015–0.023]) for the left foot. In Group DB (middle-aged adults with impairment, Table 7), the Grand Median error was 0.017 s (IQR: [0.014–0.020]) for the right foot and 0.020 s (IQR: [0.020–0.021]) for the left foot. Finally, in Group DC (young adults with impairment, Table 8), the errors were largest, with a Grand Median of 0.060 s (IQR: [0.058–0.068]) for the right foot and 0.060 s (IQR: [0.048–0.070]) for the left limb.

The corresponding median percentage errors were also noted (DA: −0.83% R, 0.74% L; DB: −2.23% R, 2.74% L; DC: −8.64% R, 1.82% L). Although precision is lower compared to the groups without impairment, the procedure demonstrates adaptability to dysfunctional gait conditions.

To evaluate the temporal foundation of our model’s segmentation, we quantified the synchronization error of the Initial Contact (IC). Table 9 presents the Median Absolute Timing Error (${\mathrm{MdAE}}_{\mathrm{Timing}}$) in milliseconds. For most groups, ${\mathrm{MdAE}}_{\mathrm{Timing}}$ remained between 60 ms and 80 ms a performance consistent with IMU systems operating at 100 Hz (10 ms per sample). This validates that the model’s event detection remains robust even under gait deterioration, which is a crucial aspect for reliable segmentation. The higher errors observed in Groups A and DC are related to the inherent difficulty of identifying IC in very soft or highly irregular gait patterns.

### 5.3. Step Length Estimation

Table 10, Table 11 and Table 12 show the step length estimation errors for Groups A (over 60 years), B (between 40 and 59 years), and C (between 18 and 39 years), respectively.

Given that the error distributions were found to be non-normal (Shapiro-Wilk test, *p* < 0.001), results are reported using non-parametric statistics: Median Absolute Error (MdAE) and Interquartile Range (IQR). The proposed model demonstrates consistent performance with low Median Absolute Errors across most subjects.

In Group A (Table 10), the grand median error was 0.027 m for the right foot (IQR: [0.022–0.034] m) and 0.034 m for the left foot (IQR: [0.028–0.039] m), indicating good estimation consistency. In Group B (Table 11), the grand median errors were 0.037 m (right, IQR: [0.023–0.059] m) and 0.048 m (left, IQR: [0.039–0.052] m), suggesting stable and reliable estimation. Finally, Group C (Table 12) showed low errors, with grand median values of 0.023 m for the right foot (IQR: [0.022–0.059] m) and 0.041 m for the left foot (IQR: [0.029–0.057] m), indicating adequate performance among young adults.

Table 13, Table 14 and Table 15 present the step length estimation errors for the groups with gait dysfunction (DA, DB, and DC), reported using robust non-parametric metrics: Median Absolute Error (MdAE) and Interquartile Range (IQR).

In Group DA (Table 13), the grand median error was 0.034 m for the right foot (IQR: [0.028–0.038] m) and 0.031 m for the left foot (IQR: [0.021–0.042] m), indicating stable performance. In Group DB (Table 14), the grand median errors were 0.055 m (right, IQR: [0.036–0.060] m) and 0.062 m (left, IQR: [0.058–0.068] m), showing slightly greater variability.

Finally, Group DC (Table 15), composed of young adults with dysfunction, exhibited low errors, with grand median values of 0.027 m for the right foot (IQR: [0.017–0.035] m) and 0.042 m for the left foot (IQR: [0.028–0.052] m), demonstrating that the model retains good estimation capability.

**Figure 10:**
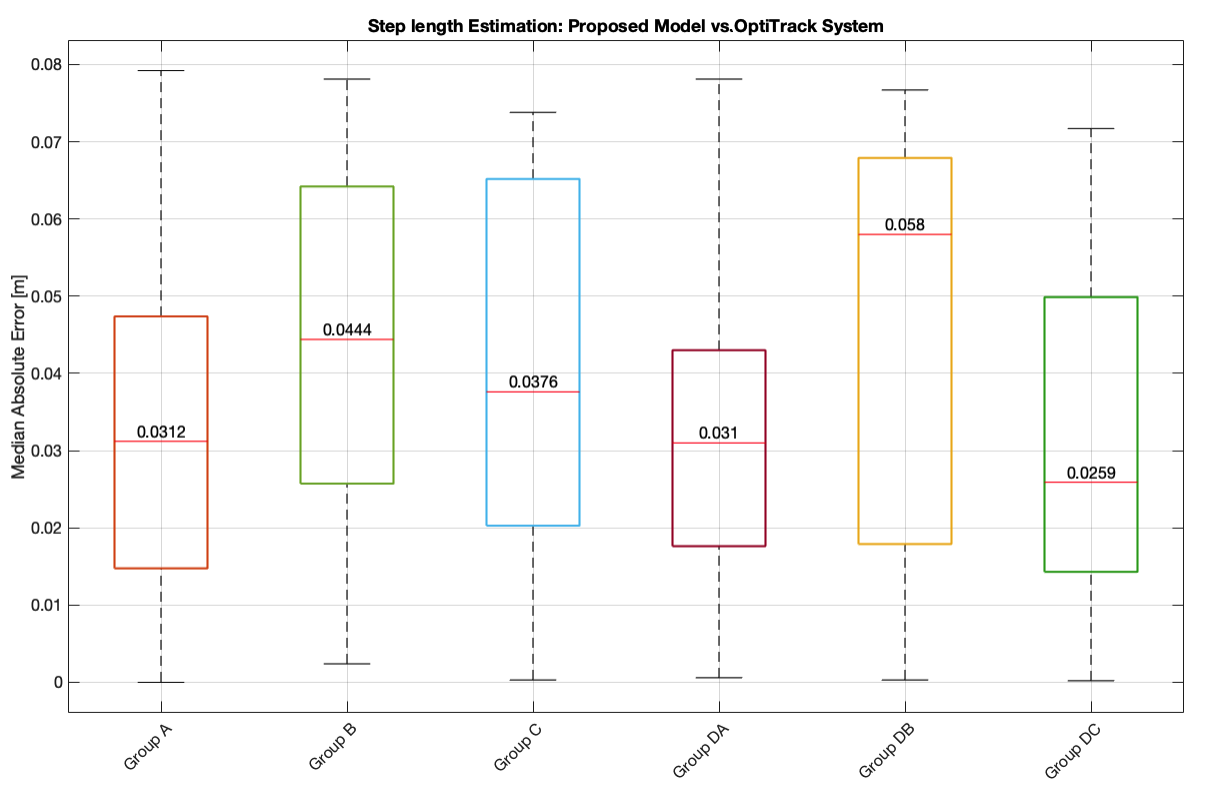
Step length estimation: Proposed Model vs. OptiTrack^®^ System.

Considering all six groups, the proposed model’s performance was evaluated using the Median Absolute Error (MdAE) (Figure 10). The analysis of the Grand Median errors (Table 10, Table 11, Table 12, Table 13, Table 14 and Table 15) shows consistent error magnitudes across all groups.

In the groups without impairment (A, B, and C), the Grand Median errors were low, ranging from 0.023 m (Group C, right) to 0.048 m (Group B, left). In the groups with gait impairment (DA, DB, and DC), the Grand Median errors remained comparable, ranging from 0.027 m (Group DC, right) to 0.062 m (Group DB, left).

These results indicate that the model maintains robust precision across all groups, with no marked differences between participants with and without gait dysfunction. Furthermore, the fact that the vast majority of median errors remain low highlights the stability of the proposed model for estimating step length under varying gait conditions.

### 5.4. Model Validation for Step Length Estimation

A Shapiro-Wilk test was first performed on the step-length error (IMU vs. OptiTrack) to assess the assumption of normality. The test confirmed that the error distribution was non-normal (*p* < 0.001), justifying the use of a non-parametric approach. Therefore, a validation analysis using the Wilcoxon signed-rank test was conducted to compare the step length estimates obtained with the proposed model against the values measured by the OptiTrack^®^ system. Due to the non-normal nature of the data, all error metrics in the results tables are reported using Median and Interquartile Range (IQR) as show in Table 10, Table 11, Table 12, Table 13, Table 14 and Table 15.

**Figure 11:**
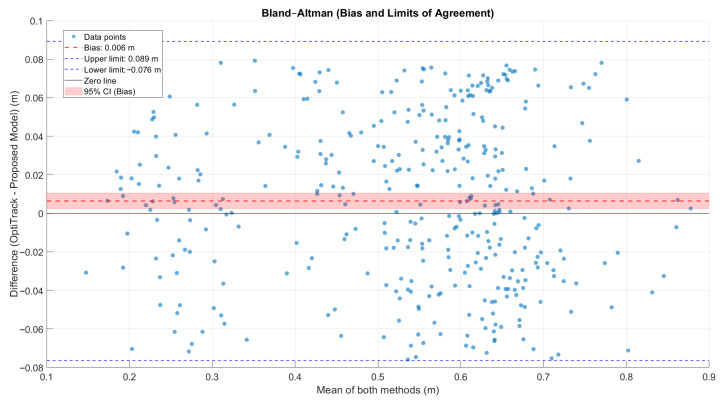
Bland–Altman plot comparing the proposed method against the OptiTrack^®^ reference system. The analysis shows a clinically trivial mean bias (0.0064 m), indicating minimal systematic error. However, the limits of agreement are wide (−0.0763 m to 0.0891 m), reflecting high random inter-step error. This suggests the method is not interchangeable for individual step analysis but is reliable for monitoring session averages due to its near-zero bias.

**Figure 12:**
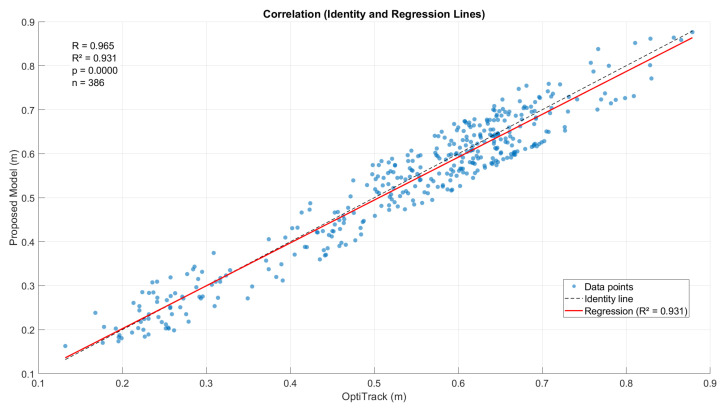
Correlation plot comparing step length estimation between the proposed model and the OptiTrack^®^ reference system. A strong linear relationship was observed (R = 0.9647, R^2^ = 0.9307, *p* < 0.001), with a regression slope of 0.9742 and an intercept of 0.0075, confirming the high consistency of the proposed model across all tested subjects.

To further assess the consistency between both measurement methods, a Bland–Altman analysis (Figure 11) and a correlation analysis (Figure 12) were performed. The Bland–Altman plot revealed a mean bias of 0.0064 m (95% CI [0.0019, 0.0104] m), with limits of agreement between −0.0763 m and 0.0891 m, resulting in an overall agreement range of 0.1655 m. The correlation analysis confirmed a strong linear relationship (R = 0.9647, R^2^ = 0.9307, *p* < 0.001), with a regression slope of 0.9742 and an intercept of 0.0075. These findings demonstrate that the proposed model accurately reproduces step length measurements with minimal systematic bias and high consistency across all tested subjects.

Furthermore, separate analyses conducted for the groups with and without gait dysfunction revealed comparable levels of agreement. In healthy subjects, the bias was 0.0091 m (95% CI [0.0036, 0.0147] m) with limits of agreement between −0.0732 m and 0.0914 m (R = 0.9166, R^2^ = 0.8402). For subjects with gait dysfunction, the bias was slightly smaller (0.0034 m, 95% CI [−0.0029, 0.0091] m), with limits of agreement from −0.0797 m to 0.0864 m (R = 0.9701, R^2^ = 0.9410). These results indicate that the proposed model maintains a high degree of accuracy and robustness even under asymmetric or irregular gait conditions.

To evaluate the robustness and generalization capacity of our method, we compared it with two interpretable Machine Learning (ML) approaches: Linear Regression and Random Forest (RF) (Table 16). Both models were trained using the following features: step duration; RMS value, mean, minimum, maximum, and peak-to-peak amplitude of each step for both the acceleration signal in the z-axis and the yaw signal.

Crucially, the ML models were evaluated using a rigorous subject-independent K-fold cross-validation protocol, ensuring that the test set consisted of subjects completely unseen during training. The results of this comparison suggest that our kinematic model significantly outperformed the simple data-driven approaches across all cohorts. In the combined dataset, our model maintained an MdAE of 0.034 m, outperforming Linear Regression (MdAE of 0.064 m) and Random Forest (MdAE of 0.066 m). Notably, the difference was most pronounced in the group with gait dysfunction. While our kinematic model maintained a high accuracy with an MdAE of only 0.031 m, both ML models showed a significant degradation in generalization to new pathological subjects: Linear Regression yielded an MdAE of 0.126 m, and Random Forest yielded 0.130 m. On this dataset, these findings strongly suggest that the explicit biomechanical structure of our model, which integrates the physical constraint of pelvic rotation, provides accuracy and robustness superior to these data-driven baselines, particularly in pathological cohorts.

On the other hand, when comparing our performance with the literature (Table 17), we observe that our accuracy is highly competitive. With an MdAE of 0.037 m for subjects without impairment, our results compare favorably with the RMSE errors of 0.06 m reported by other studies in pathological cohorts. Nevertheless, the most significant finding—and the main strength of the model—is its robustness under complex clinical conditions: the MdAE for the cohort with gait dysfunction was only 0.031 m.

## 6. Discussion

This study presents and validates an inverted double pendulum-inspired model that contributes to a meaningful advancement in gait analysis with a single inertial sensor. Its principal contribution is not merely the accurate measurement of asymmetry, but the ability to decompose it into its core biomechanical components: pendular motion and pelvic rotation. We demonstrate that isolating the influence of pelvic dynamics is crucial, transforming the model from a simple measurement tool into an interpretive framework capable of uncovering the strategies behind pathological gait, a substantial improvement beyond traditional models that assume symmetry.

The foundation of our results lies in the robust detection of gait events across all groups. In participants without impairment, errors in step duration estimation were minimal (<2%), indicating stable and unbiased detection of initial contacts. This segmentation performance, which aligns with previous work highlighting the reliability of IMU signals [29], is the foundation upon which the final step length accuracy is built.

Building on this foundation, our step length results demonstrate the model’s high fidelity and robustness across a wide range of gait conditions. The accuracy was remarkable, with low Median Absolute Errors (MdAE) across all groups. As shown in our results (Table 10, Table 11 and Table 12), the Grand Median error among older adults (Group A) was 0.027 m (IQR: [0.022–0.034] m), in middle-aged adults (Group B) it was 0.037 m (IQR: [0.023–0.059] m), and in young adults (Group C) it was 0.023 m (IQR: [0.022–0.059] m), showing that the model performs consistently across age groups.

These results, combined with percentage errors below 5%, validate the model’s effectiveness and position it favorably compared with traditional double integration methods (RMSE~8 cm [17]), approaching the accuracy of multi-sensor systems (RMSE~4 cm [18]). The statistical validation via Bland–Altman analysis revealed our most critical finding: a clinically trivial mean bias of 0.0064 m (95% CI [0.0019, 0.0104] m). However, the Limits of Agreement (LoA) were wide (−0.0763 m to 0.0891 m). This indicates that while the model is extremely accurate on average (low bias), it possesses significant random inter-step error (wide LoA). As we argue below, this means the method is not interchangeable for individual step analysis but is ideal for monitoring session averages. These findings indicate that the explicit incorporation of pelvic rotation and the use of the Froude number with the OFDRI method contribute to reducing integration drift and improving estimation stability under unconstrained gait conditions.

The value of our approach must be interpreted within the framework of the trade-off between accuracy and complexity. Although purely predictive methods—such as empirical or machine learning (ML) models—can achieve similar numerical accuracy metrics in non-pathological gait [32], our findings suggest this equivalence is compromised under conditions of asymmetry and pathology [33]. The added biomechanical complexity in our model, through the explicit formulation of the double pendulum and pelvic rotation, is not an arbitrary increase but a necessary condition for addressing clinical asymmetry. Simpler models often fail in these populations because they rely on assumptions of symmetry or on empirical stride relationships that do not hold under instability and compensatory strategies [33]. Indeed, it has been documented that event detection and parameter estimation in simple IMU configurations are unreliable in gait patterns with low speed and short steps—typical in severe impairments [34]—and that methods such as ZUPT lose effectiveness in pathological gait [35]. The inclusion of pelvic rotation is what allows our model to avoid this fundamental limitation, ensuring robustness and accuracy within clinically meaningful ranges in impaired cohorts [29]. Moreover, this added complexity enables diagnostic interpretability, transforming the model from a simple measurement tool into a framework capable of identifying the origin of gait asymmetry. Therefore, the additional effort in the model’s formulation is justified by the substantial gain in predictive robustness in clinical populations and by its diagnostic utility. To empirically validate this justification, we evaluated the performance of our kinematic model against purely predictive approaches and the existing literature, as detailed in Table 16 and Table 17.

The results presented in Table 16 address the debate regarding the need for mechanistic models versus data-driven approaches for step-length estimation. The superiority of our kinematic model over simple Linear Regression (MdAE of 0.034 m vs. 0.064 m) and its comparable or superior performance relative to Random Forest suggest that incorporating the biomechanical structure of the double pendulum is not only conceptually sound but also practically competitive in terms of accuracy. Specifically, these results suggest that, on this dataset, the proposed biomechanical model provides accuracy and robustness comparable to or better than these simple data-driven baselines, while offering clearer biomechanical interpretability—an aspect that is crucial for clinical application and diagnostic utility.

The comparison with the literature (Table 17) shows that our model maintains competitive accuracy (MdAE of 0.037 m in healthy subjects). However, the most significant advantage appears in the pathological evaluation. Our MdAE of 0.031 m in the cohort with gait dysfunction is substantially lower than the errors reported by double-integration methods and ML models in challenging clinical settings. This low error under conditions of asymmetric or irregular gait validates the use of our model as a high-precision tool for patient monitoring, where variability and the absence of a stable gait pattern are the norm and where other methods report a significant increase in error.

However, the true value of a clinical model lies in its ability to perform reliably under pathological gait conditions. In this context, the performance observed in Group DC (young adults with impairment) was particularly revealing, showing Median Absolute Error (MdAE) of 0.027 m (IQR: [0.017–0.035] m) (Figure 10), which is comparable to that of the non-impairment groups. This finding highlights that isolated gait impairment does not compromise the biomechanical foundation of the model, demonstrating its robustness and adaptability even in the presence of mild or compensated asymmetries. The model, therefore, shows strong potential for use in clinical populations, as it retains accuracy while capturing inter-limb asymmetry that is typically lost in symmetric models.

When analyzing the interaction between gait impairment and aging, a moderate increase in error was observed, particularly in Group DB (adults aged 40–59 years with impairment), which showed a MdAE of 0.055 m (right) and 0.062 m (left). This value, although higher than in other groups, indicates that the model is slightly more sensitive to gait variability typically found in middle-aged adults with dysfunction. This observation suggests that the main source of error is not structural to the model itself but rather reflects the increased irregularity and unpredictability of gait patterns in this population. The decomposition of the model allows us to infer that this variability originates mainly from the pelvic component, whose motion becomes more inconsistent compared to age-matched healthy controls (Group B). This instability in pelvic rotation may be associated with compensatory mechanisms or reduced intersegmental coordination, leading to a small cumulative deviation in step length estimation.

This type of analysis demonstrates the true potential of our approach. The model’s value extends beyond simply providing an accurate number for step length; it functions as an interpretive tool that reveals how that final step length is achieved. By decomposing the step, it highlights the outsized influence of the pelvic component, providing a more nuanced clinical picture. It allows a clinician to understand not just the magnitude of a patient’s asymmetry, but also the stability of the compensatory strategy producing it. For instance, a subject may achieve a normal step length through excessive pelvic rotation, indicating efficient compensation, whereas another may exhibit high variability, signaling reduced motor control stability. Thus, the model not only measures performance but also offers an indirect assessment of movement coordination, making it a valuable tool for evaluating rehabilitation progress and identifying subtle alterations in gait dynamics that may precede clinical symptoms.

The demonstration that our model outperforms both simple ML baselines and existing kinematic methods implies that the step-length estimation error has been substantially reduced through a structured modeling approach. Therefore, as hypothesized, the high step-to-step variability that remains can be attributed with greater confidence to the patient’s intrinsic motor-control variability rather than to a model deficiency or instrumental artifact. This reinforces the potential utility of error variability as a biomarker of motor instability—an area we will explore in depth in future correlation analyses with clinical measures (such as the Berg Balance Scale).

The coexistence of a low Median Absolute Error (MdAE) and wide Limits of Agreement (LoA) requires a clear definition of the model’s clinical scope. The width of the LoA—an expected characteristic in pathological gait analyzed with IMUs—indicates that the accuracy for estimating individual steps is limited. However, our low MdAE validates that the approach is well suited for continuous monitoring and analysis based on aggregated parameters (such as moving medians or session averages). Beyond measurement itself, this distinction is clinically meaningful: the high step-to-step variability reflected in the LoA should not necessarily be interpreted as a reduction in model accuracy but, as hypothesized, as a potential indicator of instability or inefficiency in motor-control strategies, thereby reinforcing the model’s utility for long-term monitoring.

To address this, we propose two lines of investigation: (1) an analysis of inter-limb coordination aimed at explicitly characterizing coordination between limbs using phase relationships—for example, by applying Hilbert phase analysis to the time series of pelvic rotation (Yaw) or tilt (Roll). This would allow us to quantify how pelvic versus pendular components change with phase patterns; and (2) a Temporal Variability Structure analysis, aimed at examining the step-by-step error time series for long-range dependence or structured variability. Techniques such as Detrended Fluctuation Analysis (DFA) can determine whether the observed error corresponds to pure white noise or whether it instead exhibits a complex variability structure that better aligns with our hypothesis of biological instability.

Because the model is particularly sensitive to variations in pelvic rotation and inter-limb asymmetry, deviations in its estimations may reflect fluctuations in coordination or rhythm rather than noise. This characteristic provides valuable clinical insight, as it allows distinguishing between stable and unstable gait patterns, offering indirect information on balance control, rehabilitation progress, or even fall risk. In this way, the model transcends simple measurement, serving as a biomechanical lens to interpret the quality and consistency of human gait.

This interpretation leads us to delve into the model’s conceptual limitations. The increased error in Group DB suggests that the pendulum approach, while an excellent approximation, has its limits in pathologies with high spasticity or rigidity where the leg ceases to behave like a passive pendulum. Consequently, the current model, unable to capture these non-linear dynamics, underestimates displacement. Similarly, our analysis confirms that the entire system is critically dependent on the detection of the initial contact. In pathological gait, as demonstrated in [33], alterations in phase timing complicate automated detection [36,37]. In our case, the prevalence of soft heel strikes creates a biomechanically ambiguous event by attenuating the anteroposterior acceleration peaks. This introduces temporal uncertainty in the exact localization of the event—directly explaining the step duration variability observed in Table 8—which propagates to the step length estimation.

In this context, we acknowledge an important methodological limitation: the exclusion of recordings in which gait events were “clinically absent” (for example, in extremely shuffling gait). Although this exclusion is technically understandable due to event ambiguity, it introduces a selection bias by systematically removing the most severe pathological patterns. Therefore, although our model is robust to asymmetry, the conclusions should not be over-generalized to the most severe cases of shuffling gait, which represents a key challenge for future development

However, it is noteworthy that despite these signal challenges, the algorithm successfully detected events without the need for data exclusion. The fact that the median error remained within clinically useful ranges (<0.034 m, Table 16) suggests that the biomechanical model is robust against detection errors, offering clinically useful estimates even with the inherent challenges of pathological gait signals

Thus, the identified limitations guide the future development of the model, with the goal of turning it into a clinically comprehensive tool. We propose two lines of work: First, refinement through signal fusion and predictive filtering. To address the critical limitation—sensitivity to event detection—we propose a conceptual evolution: shifting from a purely acceleration-based detection to a signal fusion approach. Since angular velocity signals are driven by limb swing rather than ground impact, they remain robust even when heel-strike acceleration is attenuated (e.g., in shuffling gait). Therefore, future work will integrate angular velocity features into a predictive–corrective filter (e.g., an Extended Kalman Filter). Here, the biomechanical model would act as an active predictor of the next initial contact, while the multi-modal sensor signal would be used for correction.

Second, personalization of the biomechanical model. We propose investigating the adaptation of the model for specific populations. This could be achieved by adjusting the model’s coefficients through a calibration period—creating optimized parameters for patients with certain pathologies—or even by incorporating a new term into the equation to model joint stiffness. Additionally, given the heterogeneity of diagnoses in our clinical cohort, future work will explore the relationship between pathology type and the source of model error (pelvis vs. pendulum), aiming to identify biomechanical compensation patterns that may allow the model to be used as a tool for differential diagnosis.

On the other hand, the need for an individualized measurement of the pelvic radius ($R_{p}$) is identified as a practical limitation for large-scale implementation. Although in our study $R_{p}$ was measured manually for scientific rigor during model validation, this practice is unfeasible in remote or large-scale monitoring environments. Therefore, future work should focus on the development of robust anthropometric estimation of $R_{p}$. It is important to note that an estimation based solely on height (e.g., a constant scaling factor) could introduce significant systematic error, especially in cohorts with a wide range of Body Mass Index (BMI), where pelvic mass distribution directly influences $R_{p}$. Instead, we propose investigating the accuracy of multivariable regression models that incorporate a combination of easily accessible parameters (such as height, weight, and age) to provide an estimate of $R_{p}$ that is sufficiently precise to preserve the model’s clinical applicability.

Despite the biomechanical robustness demonstrated under pathological asymmetry, our model exhibits limitations inherent to IMU technology in real clinical environments. The main reliability challenge is its sensitivity to sensor misplacement and motion artifacts at L5-S1. Accurate measurements depend on the alignment between the sensor’s reference frame and the anatomical frame. A vertical-axis displacement compromises the correct kinematic representation of the Center of Mass (CM), leading to distortions in step estimation. More critically, mediolateral displacement or angular misalignment (Yaw/Roll) induces coupling of transverse and vertical accelerations into the anteroposterior signal, directly affecting step-calculation accuracy and the validity of pelvic rotation $R_{p}$. Although our results were obtained under controlled placement conditions, full clinical applicability requires overcoming this vulnerability. Therefore, future work should focus on the systematic quantification of the model’s sensitivity to misplacement and on the development of algorithms that minimize the impact of initial misalignment and belt motion, ensuring an effective transition to ambulatory monitoring.

While the measurements of the central pelvic rotation component (Yaw) of the sensor were validated in controlled laboratory settings, the model’s robustness in real-world environments with magnetic disturbances remains an untested aspect. This validation in uncontrolled environments is a necessary future step for bringing the system to large-scale clinical application.

In summary, this work validates a model that does more than accurately estimate step length; it provides a biomechanical framework to interpret the strategy behind a patient’s gait. By decomposing the step and isolating the critical role of pelvic rotation, we have shown that the model’s performance, particularly its error, can serve as a powerful clinical indicator. The low systematic bias (0.64 cm) and the model’s ability to estimate asymmetry make it a robust tool for longitudinal monitoring. The identified limitations are therefore not failures but key insights that guide the path toward models capable of capturing even more complex, non-linear dynamics, pushing forward the development of truly personalized and clinically integral tools.

## 7. Conclusions

A new biomechanical model inspired by an inverted double pendulum was presented, which, by incorporating pelvic rotations, allows for accurate step length estimation under asymmetric gait conditions using a single inertial sensor placed on the lower back. In participants without gait impairment, the method achieved Median Absolute Errors (MdAE) below 0.04 m.

Statistical validation confirmed the model’s robustness. Crucially, the Bland-Altman analysis revealed a clinically trivial systematic bias (0.0064 m). While the limits of agreement were wide (reflecting high inter-step variability), this near-zero bias demonstrates the technical feasibility of this portable system under controlled conditions and its strong potential for future implementation.

In impaired gait, the model provides a strong indication of its clinical value by consistently reflecting inter-limb asymmetry and by showing significantly greater robustness than data-driven baselines when evaluated under subject-independent cross-validation. Although the Median Absolute Error increases in certain cohorts (up to 0.062m). This sensitivity to the gait pattern confirms that the main challenge and future direction of this work is the refinement of event detection to adapt the model to the variability found in complex clinical populations.

Despite this limitation, which in turn represents a clear avenue for future research, the model is envisioned as a low-cost, portable, and versatile tool. Its extremely low systematic bias makes it unsuitable for individual step diagnosis but grants it high reliability for obtaining an accurate session average (i.e., from a complete walk test). This, in turn, grants it significant potential for implementation in home-based assessments, rehabilitation programs, and longitudinal patient monitoring, where tracking change over time is the primary goal.

## Figures and Tables

**Table 1:** Description of age groups with and without gait impairment.

	Group A	Group B	Group C	Group DA	Group DB	Group DC
Age range	≥60 years	40 to 59 years	18 to 39 years	≥60 years	40 to 59 years	18 to 39 years
N	9 women	4 men 2 women	3 men 3 women	4 women	3 men 1 woman	3 men 1 woman
Age (years)	69.47 ± 5.46	49.8 ± 5	26 ± 6.55	73 ± 4.24	51.75 ± 5.85	26 ± 3.74
Weight (kg)	73.33 ± 14.54	81.33 ± 13.61	74.88 ± 16.97	69.5 ± 9.84	70 ± 12.52	68.35 ± 7.31
Height (m)	1.54 ± 0.06	1.65 ± 0.11	1.65 ± 0.09	1.49 ± 0.08	1.61 ± 0.03	1.69 ± 0.1
Right leg length (m)	0.84 ± 0.045	0.87 ± 0.056	0.90 ± 0.041	0.86 ± 0.053	0.88 ± 0.038	0.94 ± 0.056
Left leg length (m)	0.84 ± 0.041	0.88 ± 0.062	0.90 ± 0.042	0.84 ± 0.056	0.87 ± 0.035	0.94 ± 0.051

**Table 2:** Characteristics of the Inertial and Magnetic Sensors of the IMU to be used.

Sensor	Axis	Range	Bandwidth	Resolution	Frequency
Accelerometer	XYZ	±16 G	62.5 Hz	14 bits (~1.95 mG)	100 Hz
Gyroscope	XYZ	±2000 dps	32 Hz	16 bits (~0.061 dps)	100 Hz
Magnetometer	XY	±1300 [μT]	10 Hz	13 bits (~317 [ηT])	20 Hz
	Z	±2500 [μT]	10 Hz	15 bits (~152 [ηT])	20 Hz

**Table 3:** Step duration error Group A.

Subject	Step Duration Right			Step Duration Left		
	MdAE [s]	IQR [s]	% Error	MdAE [s]	IQR [s]	% Error
A01	0.010	[0–0.020]	−1.69	0.035	[0.015–0.040]	3.86
A02	0.015	[0.010–0.038]	−1.88	0.01	[0.010–0.010]	1.92
A03	0.003	[0.003–0.013]	0.71	0.003	[0.003–0.005]	−0.68
A04	0.015	[0.008–0.029]	0.14	0.018	[0.013–0.031]	2.60
A05	0.005	[0.003–0.009]	−0.31	0.010	[0.007–0.016]	1.39
A06	0.020	[0.013–0.025]	−3.47	0.029	[0.022–0.035]	5.59
A07	0.020	[0.010–0.020]	3.33	0.010	[0.008–0.028]	−1.65
A08	0.015	[0.003–0.043]	−1.02	0.010	[0.003–0.010]	1.04
A09	0.010	[0.003–0.010]	0	0	[0–0]	0
Grand Median	0.015	[0.010–0.018]	−0.31	0.010	[0.010–0.024]	1.39

Error metrics for right and left step duration for each of the nine subjects, including the median and IQR (in seconds) and the percent error. The grand median across all participants is also provided.

**Table 4:** Step duration error Group B.

Subject	Step Duration Right			Step Duration Left		
	MdAE [s]	IQR [s]	% Error	MdAE [s]	IQR [s]	% Error
B01	0.015	[0.010–0.020]	1.96	0.010	[0–0.020]	0
B02	0.010	[0.008–0.013]	1.80	0.010	[0.005–0.010]	0
B03	0.020	[0.010–0.020]	−3.70	0.020	[0.010–0.020]	3.77
B04	0.010	[0.005–0.040]	0	0.010	[0.010–0.015]	1.88
B05	0.015	[0.008–0.035]	−0.94	0.015	[0.010–0.028]	−2.79
B06	0.010	[0.010–0.010]	1.85	0.010	[0.010–0.040]	−1.75
Grand Median	0.012	[0.010–0.015]	0.90	0.010	[0.010–0.015]	0

Error metrics for right and left step duration for each of the six subjects, including the median and IQR (in seconds) and the percent error. The grand median across all participants is also provided.

**Table 5:** Step duration error Group C.

Subject	Step Duration Right			Step Duration Left		
	MdAE [s]	IQR [s]	% Error	MdAE [s]	IQR [s]	% Error
C01	0.020	[0.010–0.020]	1.75	0.010	[0.010–0.013]	1.78
C02	0	[0–0.003]	0	0.010	[0.010–0.030]	1.88
C03	0.010	[0–0.010]	1.81	0	[0–0]	0
C04	0.010	[0.008–0.010]	−0.90	0.020	[0.010–0.030]	1.81
C05	0.060	[0.035–0.065]	−9.83	0.025	[0.008–0.043]	3.99
C06	0.050	[0.040–0.055]	8.55	0.060	[0.050–0.130]	−10.16
Grand Median	0.015	[0.010–0.050]	0.87	0.015	[0.010–0.025]	1.80

Error metrics for right and left step duration for each of the six subjects, including the median and IQR (in seconds) and the percent error. The grand median across all participants is also provided.

**Table 6:** Step duration error Group DA.

Subject	Step Duration Right			Step Duration Left		
	MdAE [s]	IQR [s]	% Error	MdAE [s]	IQR [s]	% Error
DA01	0.010	[0.010–0.040]	−1.72	0.010	[0.010–0.010]	0
DA02	0.030	[0.010–0.035]	−1.66	0.025	[0.020–0.038]	3.36
DA03	0.010	[0.003–0.020]	0	0.020	[0.010–0.038]	1.48
DA04	0.030	[0.015–0.085]	1.85	0.020	[0.010–0.050]	−1.75
Grand Median	0.020	[0.010–0.030]	−0.83	0.020	[0.015–0.023]	0.74

Error metrics for right and left step duration for each of the four subjects, including the median and IQR (in seconds) and the percent error. The grand median across all participants is also provided.

**Table 7:** Step duration error Group DB.

Subject	Step Duration Right			Step Duration Left		
	MdAE [s]	IQR [s]	% Error	MdAE [s]	IQR [s]	% Error
DB01	0.015	[0.010–0.050]	−2.79	0.025	[0.020–0.033]	4.59
DB02	0.020	[0.010–0.035]	−1.68	0.020	[0.010–0.040]	3.63
DB03	0.010	[0.010–0.040]	1.85	0.020	[0.010–0.020]	−3.77
DB04	0.020	[0.010–0.030]	−3.70	0.020	[0.010–0.030]	1.85
Grand Median	0.017	[0.014–0.020]	−2.23	0.020	[0.020–0.021]	2.74

Error metrics for right and left step duration for each of the four subjects, including the median and IQR (in seconds) and the percent error. The grand median across all participants is also provided.

**Table 8:** Step duration error Group DC.

Subject	Step Duration Right			Step Duration Left		
	MdAE [s]	IQR [s]	% Error	MdAE [s]	IQR [s]	% Error
DC01	0.090	[0.080–0.100]	−17.39	0.070	[0.045–0.125]	−8.01
DC02	0.060	[0.030–0.070]	8.82	0.040	[0.018–0.065]	−3.49
DC03	0.060	[0.060–0.070]	−10	0.070	[0.040–0.090]	7.14
DC04	0.050	[0.033–0.060]	−7.29	0.050	[0.040–0.060]	7.24
Grand Median	0.060	[0.058–0.068]	−8.64	0.060	[0.048–0.070]	1.82

Error metrics for right and left step duration for each of the four subjects, including the median and IQR (in seconds) and the percent error. The grand median across all participants is also provided.

**Table 9:** Median Absolute Error (MdAE) of Initial Contact (IC) Timing Synchronization (${\mathrm{MdAE}}_{\mathrm{Timing}}$) versus OptiTrack^®^.

Group	Synchronization Right Limb		Synchronization Left Limb	
	MdAE [ms]	IQR [ms]	MdAE [ms]	IQR [ms]
Group A	100	[40–63]	110	[50–150]
Group B	60	[40–80]	65	[40–90]
Group C	80	[30–100]	75	[40–110]
Group DA	60	[30–100]	70	[20–113]
Group DB	55	[20–160]	30	[10–80]
Group DC	110	[50–163]	90	[30–90]

**Table 10:** Step length Error Group A.

Subject	MdAE Right			MdAE Left		
	Median [m]	IQR [m]	% Error	Median [m]	IQR [m]	% Error
A01	0.033	[0.006–0.039]	0.02	0.042	[0.022–0.064]	4.54
A02	0.023	[0.010–0.032]	1.70	0.038	[0.011–0.041]	5.91
A03	0.009	[0.004–0.034]	−1.45	0.029	[0.018–0.061]	−3.86
A04	0.031	[0.024–0.038]	−6.55	0.048	[0.032–0.061]	−11.95
A05	0.052	[0.044–0.053]	−9.01	0.034	[0.014–0.043]	3.41
A06	0.037	[0.028–0.048]	−1.03	0.038	[0.008–0.062]	−4.45
A07	0.019	[0.016–0.034]	−3.03	0.026	[0.007–0.042]	3.10
A08	0.027	[0.004–0.052]	−2.55	0.028	[0.015–0.037]	0.16
A09	0.026	[0.005–0.041]	−2.80	0.020	[0.017–0.034]	−0.82
Grand Median	0.027	[0.022–0.034]	−2.80	0.034	[0.028–0.039]	0.16

Median absolute error metrics for right and left step length for each of the nine subjects, including the median and interquartile range (IQR) (in meters) and the percent error. The grand median and IQR across all participants is also provided.

**Table 11:** Step length Error Group B.

Subject	MdAE Right			MdAE Left		
	Median [m]	IQR [m]	% Error	Median [m]	IQR [m]	% Error
B01	0.023	[0.009–0.039]	−3.62	0.055	[0.046–0.066]	−8.05
B02	0.023	[0.019–0.046]	0.10	0.046	[0.036–0.070]	−6.07
B03	0.032	[0.016–0.046]	−5.76	0.052	[0.049–0.070]	7.45
B04	0.041	[0.015–0.063]	0.84	0.027	[0.008–0.051]	−3.28
B05	0.059	[0.028–0.072]	−8.71	0.049	[0.027–0.070]	−3.12
B06	0.060	[0.033–0.065]	−9.44	0.039	[0.019–0.064]	−6.71
Grand Median	0.037	[0.023–0.059]	−4.69	0.048	[0.039–0.052]	−4.68

Median absolute error metrics for right and left step length for each of the six subjects, including the median and interquartile range (IQR) (in meters) and the percent error. The grand median and IQR across all participants is also provided.

**Table 12:** Step length Error Group C.

Subject	MdAE Right			MdAE Left		
	Median [m]	IQR [m]	% Error	Median [m]	IQR [m]	% Error
C01	0.023	[0.014–0.073]	−0.27	0.017	[0.009–0.036]	2.09
C02	0.022	[0.011–0.050]	−1.76	0.046	[0.029–0.062]	6.83
C03	0.022	[0.005–0.067]	0.04	0.037	[0.011–0.081]	6.38
C04	0.016	[0.005–0.027]	−0.80	0.029	[0.021–0.060]	−3.32
C05	0.072	[0.046–0.074]	−8.26	0.057	[0.041–0.068]	0.83
C06	0.059	[0.037–0.073]	8.76	0.061	[0.038–0.071]	3.79
Grand Median	0.023	[0.022–0.059]	−0.54	0.041	[0.029–0.057]	2.94

Median absolute error metrics for right and left step length for each of the six subjects, including the median and interquartile range (IQR) (in meters) and the percent error. The grand median and IQR across all participants is also provided.

**Table 13:** Step length Error Group DA.

Subject	MdAE Right			MdAE Left		
	Median [m]	IQR [m]	% Error	Median [m]	IQR [m]	% Error
DA01	0.039	[0.011–0.050]	1.92	0.022	[0.020–0.043]	3.40
DA02	0.030	[0.027–0.057]	−4.59	0.045	[0.037–0.075]	5.25
DA03	0.026	[0.014–0.033]	−6.63	0.020	[0.006–0.041]	−2.68
DA04	0.038	[0.013–0.047]	6.96	0.040	[0.036–0.057]	6.73
Grand Median	0.034	[0.028–0.038]	−1.33	0.031	[0.021–0.042]	4.32

Median absolute error metrics for right and left step length for each of the four subjects, including the median and interquartile range (IQR) (in meters) and the percent error. The grand median and IQR across all participants is also provided.

**Table 14:** Step length Error Group DB.

Subject	MdAE Right			MdAE Left		
	Median [m]	IQR [m]	% Error	Median [m]	IQR [m]	% Error
DB01	0.056	[0.010–0.083]	−6.08	0.063	[0.021–0.072]	−10.49
DB02	0.065	[0.033–0.069]	−9.54	0.060	[0.020–0.068]	−9.50
DB03	0.018	[0.001–0.041]	−0.18	0.055	[0.020–0.060]	0.04
DB04	0.055	[0.027–0.064]	−6.52	0.072	[0.052–0.084]	4.98
Grand Median	0.055	[0.036–0.060]	−6.30	0.062	[0.058–0.068]	−4.73

Median absolute error metrics for right and left step length for each of the four subjects, including the median and interquartile range (IQR) (in meters) and the percent error. The grand median and IQR across all participants is also provided.

**Table 15:** Step length Error Group DC.

Subject	MdAE Right			MdAE Left		
	Median [m]	IQR [m]	% Error	Median [m]	IQR [m]	% Error
DC01	0.015	[0.014–0.030]	2.95	0.038	[0.022–0.071]	3.01
DC02	0.035	[0.020–0.047]	−4.70	0.058	[0.027–0.075]	−8.64
DC03	0.036	[0.034–0.049]	−5.08	0.018	[0.008–0.024]	1.64
DC04	0.019	[0.010–0.047]	−0.72	0.047	[0.016–0.058]	17.74
Grand Median	0.027	[0.017–0.035]	−2.71	0.042	[0.028–0.052]	2.32

Median absolute error metrics for right and left step length for each of the four subjects, including the median and interquartile range (IQR) (in meters) and the percent error. The grand median and IQR across all participants is also provided.

**Table 16:** Performance Evaluation: Proposed Model vs. Predictive Methods (ML) for Step length estimation.

Method	MdAE All Subjects	MdAE Unimpaired Gait	MdAE Impaired Gait
Our Model	0.034 m	0.037 m	0.031 m
Lineal Regression	0.064 m	0.067 m	0.126 m
Random Forest	0.066 m	0.068 m	0.130 m

MdAE: Median Absolute Error.

**Table 17:** Comparison of Step Length Estimation Performance using Single lumbar IMUs in Pathological and Non-Pathological Cohorts.

Authors	Model/Algorithm	Sensor Location	Participants	Reported Error
Our Model	Inverted double-pendulum (simple integration)	1 IMU (L5-S1)	Subjects with and without gait impairment (33)	MdAE 0.037 m (unimpaired gait) 0.031 m (impaired gait)
Soltani [17]	Inverted pendulum (Double integration with corrective filtering)	1 IMU (L4-S2)	Subjects with and without gait impairment (40)	RMSE:0.04 m (Slow speed)0.08 m (Normal speed)0.07 m (Fast speed)0.17 m (Assistive device)
Zadka [31]	Machine Learning (XGBoost)	1 IMU (L5-S1)	Young and older adults with different neurological conditions (472)	RMSE 0.060 m (single step) 0.047 m (10-step average)

MdAE: Median Absolute Error; RMSE: Root Mean Square Error.

## Data Availability

The datasets presented in this article are not readily available because the data are part of an ongoing study.
